# Secondary Focal Segmental Glomerulosclerosis: From Podocyte Injury to Glomerulosclerosis

**DOI:** 10.1155/2016/1630365

**Published:** 2016-03-21

**Authors:** Jae Seok Kim, Byoung Geun Han, Seung Ok Choi, Seung-Kuy Cha

**Affiliations:** ^1^Division of Nephrology, Department of Internal Medicine, Yonsei University Wonju College of Medicine, Wonju 220-701, Republic of Korea; ^2^Departments of Physiology and Global Medical Science, Yonsei University Wonju College of Medicine, Wonju 220-701, Republic of Korea

## Abstract

Focal segmental glomerulosclerosis (FSGS) is a common cause of proteinuria and nephrotic syndrome leading to end stage renal disease (ESRD). There are two types of FSGS, primary (idiopathic) and secondary forms. Secondary FSGS shows less severe clinical features compared to those of the primary one. However, secondary FSGS has an important clinical significance because a variety of renal diseases progress to ESRD thorough the form of secondary FSGS. The defining feature of FSGS is proteinuria. The key event of FSGS is podocyte injury which is caused by multiple factors. Unanswered questions about how these factors act on podocytes to cause secondary FSGS are various and ill-defined. In this review, we provide brief overview and new insights into FSGS, podocyte injury, and their potential linkage suggesting clues to answer for treatment of the disease.

## 1. Introduction

Focal segmental glomerulosclerosis (FSGS) is now considered as a group of clinicopathological syndromes sharing a common histologic lesion characterized by focal and segmental scarring in glomerulus. Although a variety of factors could cause FSGS, the common pathogenic mechanism is podocyte injury. FSGS and a related disorder, minimal change disease, are so called “podocytopathy” [1] whose primary pathologic feature is effacement of the podocyte foot processes. Podocyte (visceral epithelium) is a unique terminally differentiated cell providing the permselectivity for a glomerular filtration barrier. Interdigitating processes of podocyte covering glomerular capillaries develop slits to function as gatekeeper for kidney filtration. Structural changes in podocyte result from podocyte injury, which leads to podocyte loss. Podocytopenia is a major event in the beginning of glomerulosclerosis.

There are two types of FSGS, primary (idiopathic) and secondary forms. The specific cause of primary FSGS has been ill-defined. Recently, clinical evidence suggested that primary FSGS is associated with causative circulating permeable factors including soluble urokinase plasminogen activator receptor (suPAR), although definite cause is not yet documented [2, 3]. Primary FSGS is a representative disorder presenting nephrotic syndrome and is a major type of primary glomerulonephritis [4] and accounts for 4% of end stage renal disease (ESRD) in the United States [5]. In comparison, secondary FSGS often presents with nonnephrotic proteinuria and less clinical severity. Nevertheless, secondary FSGS still has clinical significance; most cases of secondary FSGS are consequences from renal adaptive processes in a variety of renal diseases. Therefore, understanding about secondary FSGS provides clue to how podocyte and glomerulus adapt to renal injury and survive. Here, we review the pathogenic mechanisms underlying secondary FSGS focused on the podocyte injury causing foot process effacement and glomerulosclerosis.

## 2. Podocyte Injury and Glomerulosclerosis

### 2.1. Structure of Podocyte and Actin Cytoskeleton

A large body of studies describe the structures and physiologic roles of podocyte supporting the fact that podocyte is dynamic [6]. Podocyte contains coordinated systems composed of contractile cytoskeletal fibers and associated proteins [7] including actin, myosin II, synaptopodin, talin, vinculin, and *α*-actinin-4. These systems are critical for maintaining the integrity of podocyte against pathological microenvironmental changes in the glomerulus. Actin cytoskeleton especially plays a major role in maintaining foot process function via integrating all structural components [8, 9], and actin rearrangement is common pathway to develop foot process effacement no matter what causes podocyte injury [10]. The actin cytoskeleton is connected to apical, lateral, and basal areas of podocyte to maintain cooperation between them [11] suggesting that optimal spatial organization of cytoskeleton is crucial for podocyte function. Each area of podocyte is composed of diverse interacting proteins which maintain cell to cell and cell to glomerular basement membrane (GBM) contacts and sense mechanical changes from outer environment to deliver them to the actin cytoskeleton [12, 13].

### 2.2. Is Podocyte a Major Player to Counterbalance Capillary Distending Pressure?

The podocyte foot processes essentially provide the glomerular filtration barrier to filter plasma through slits and also have a tensile strength to oppose capillary distending pressure [14]. But several observations argued that the attribution of podocyte to oppose the hydraulic pressure from capillary was minor because podocyte did not encircle capillary completely [15]. This structural limitation demonstrates that podocyte does not provide enough opposite strength, and integrin connections in basal side of podocyte have a limited role in fixing only individual podocyte to the GBM. Instead, GBM and mesangial cell play a major role in counteracting and balancing the capillary distending pressure [15]. The GBM has basically elastic structure to endure the distending stress and is able to increase resistant force by reinforcing elastic structures according to rise in capillary pressure. Mesangial cell also counterbalances capillary pressures by supporting connections with the GBM and by cell contraction [15].

### 2.3. Is Foot Process Effacement an Adaptive Process or Just a Result of Disruption of Integrated System?

Even though the pathogenic mechanism of foot process effacement has been suggested, it is unclear whether the foot process effacement is an adaptive process to podocyte injury or is merely the result of disintegration of highly organized system. Multiple studies suggested that podocyte responded to mechanical stress originated from capillary pressure [14] and that foot process effacement might be an adaptive mechanism to increase the capability of attachment to GBM against increased capillary pressure. Furthermore, several observations demonstrated that foot process effacement was reversible [16] supporting the adaptive role of foot process effacement. On the other hand, foot process effacement could be induced by nonmechanical injury [9, 17, 18] or via unknown mechanism. Genetic mutations leading to foot process effacement do not seem to have a relationship with an adaptive process [19]. Rather, it is suggested that foot process effacement might be just a result of the disruption of integrated system to maintain the shape of foot process.

### 2.4. How Does Foot Process Effacement Progress?

**Figure 1:**
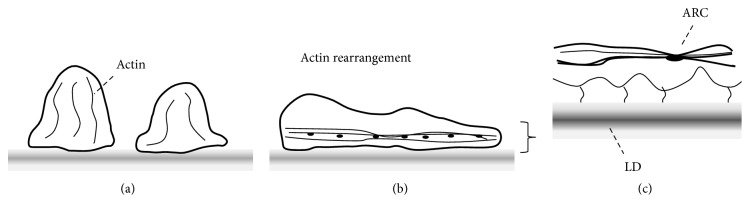
Rearrangement of actin cytoskeletons. (a) Podocyte foot processes and actin cytoskeletons in physiologic condition. (b) Actin cytoskeletons are rearranged into dense network at the basal area of foot process with effacement. (c) Actin-rich center (ARC) is formed within the dense network of actin cytoskeleton to maintain the network. Microfilaments are connected between basal side of foot process and lamina densa (LD) of glomerular basement membrane.

Multiple studies described ultrastructural findings related to foot process effacement. The universal finding is rearrangement of actin cytoskeleton of podocyte leading to dense network. It is believed that the actin rearrangement is the common cause leading to foot process effacement [10, 20]. Shirato et al. described that, in progress of foot process effacement, the actin cytoskeleton was remodeled to form microfilamentous mat at the sole of podocyte and regular dense bodies within the microfilamentous network served as cross-linker to maintain the dense network. In addition, the surface of effaced process facing GBM had irregular shape, and the dense microfilamentous cytoskeletons connected basal surface of podocyte with lamina densa of GBM. As a result, foot process effacement reinforced the ability of podocyte to counteract the distending forces of capillary [16]. Endlich et al. also reported similar findings. They demonstrated that podocyte processes were thinner and more elongated against a mechanical stress* in vitro.* Stress fibers in podocyte were rearranged from transversal shape into radial shape and actin-rich centers which were described as dense bodies in Shirato's report increased in number and size (Figure 1) [21]. Additionally, it should be noted that molecular compositions of a slit diaphragm can be altered without visible changes in morphology, and foot process structures are reorganized to close filtration slits and to displace the slit diaphragm apically, in early phase of podocyte injury [9, 11]. In an elegant review by Mundel and Shankland, four major causes leading to foot process effacement were suggested: (1) interference with the slit diaphragm complex and its lipid rafts, (2) interference with the GBM or the podocyte-GBM interaction, (3) interference with the actin cytoskeleton and its associated protein *α*-actinin-4, and (4) interference with the negative apical membrane domain of podocytes (e.g., neutralization of negative cell surface charges) [9, 11]. Overall, the actin cytoskeleton remodeling initiated by either mechanical or nonmechanical stress is probably an important general pathogenic mechanism for foot process effacement of podocyte involving the attachment of podocyte to GBM.

### 2.5. Podocytopenia Is an Early Event of Glomerulosclerosis

Podocyte has no proliferative potential as a terminally differentiated cell. Therefore, loss of podocyte is not replaced by new podocyte leading to podocytopenia. Podocytopenia is associated with renal outcomes such as increased proteinuria, glomerulosclerosis, and renal disease progression [22]. In addition, clinical nephropathy is closely related with the pathognomonic findings such as glomerulomegaly, mesangial expansion, broadened podocytes, and less number of podocytes than those with normoalbuminuria or microalbuminuria [23]. Consistent with these observations, several studies support the notion that loss of podocyte is positively correlated with the extent of albuminuria, glomerulosclerosis, and disease severity in patients with IgA nephropathy [24] as well as in a puromycin aminonucleoside (PAN) nephropathy [25]. In early stage of FSGS, cellular lesions including transformed podocytes were accompanied by segmental sclerosis. This observation supports the fact that podocyte damage might be an early event of glomerulosclerosis [26]. Rennke suggested a unique paradigm of glomerulosclerosis development summarized as follows: (1) podocyte injury, (2) foot process effacement and podocyte hypertrophy, (3) endothelial-mesangial hyperplasia and glomerulomegaly, (4) loss of podocytes and denudation of GBM, (5) increased nonselective filtration flow through bare areas of GBM, (6) collapse and occlusion of capillary loops by macromolecules in filtrate on bare areas of GBM, and (7) disruption of glomerular tuft and adhesion to Bowman's capsule [27]. In recent elegant reviews, a novel concept and the essential steps of glomerulosclerosis were suggested as follows: (1) increased glomerular capillary pressure and filtration flow through podocyte slits, (2) foot process effacement as an adaptive response, (3) podocyte hypertrophy and glomerulomegaly, (4) mismatch between glomerular tuft growth and podocyte hypertrophy, (5) stretching and attenuation of podocyte cell body, (6) pseudocysts formation by hindered flow of filtrates beneath the podocyte that is partially detached on bare areas of GBM, (7) complete podocyte detachment by enlarged pseudocysts and adhesion to Bowman's capsule, (8) glomerular tuft's adhesion to Bowman's capsule, (9) spreading of filtrates to interstitium out of nephron through adhesion structure, and (10) interstitial proliferation and nephron degeneration [15, 28–30].

In classic view, podocytes are terminally differentiated cell and have weak motility causing podocytopenia responding to glomerular injury. However, podocytes can be proliferated [2] and replaced by parietal epithelial cells (PECs), which serve as podocyte progenitor [31, 32]. Recently, new paradigms including PECs shed light on glomerular physiology and glomerular diseases [31–36]. PECs exert protective role which responded to podocyte depletion via their progenitor function. Conversely, it has been also suggested that PECs contributed pathological role in the formation of sclerotic lesion in FSGS [33, 34]. The PECs were previously known to be included in the process of glomerular crescents [35]. Similarly, the activated PECs induced adhesion between denuded GBM of tuft and Bowman's capsule in the process of glomerular sclerosis. Then the activated PECs invaded the affected glomerular tuft and increased extracellular matrix leading to glomerular sclerosis [36], suggesting that glomerular sclerosis by activated PECs may represent the active process to prevent further functional deficit beyond the passive result to injury. Cumulated studies argue whether PECs protect podocytopenia via their progenitor function or contribute to glomerular pathology including crescent formation and extracellular matrix accumulation. The selective targeting to the progenitor function of PECs responding to podocyte depletion may provide clues to treatment of the podocytopenia.

### 2.6. Foot Process Effacement Is the Instinct for Survival

**Figure 2:**
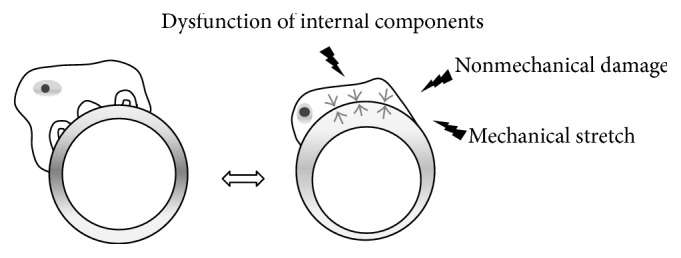
The shape of podocyte is changed with foot process effacement when mechanical or nonmechanical stresses are given or internal components are disrupted.

Podocyte detachment is the final destiny of podocyte injury, although the dropped out podocyte is still viable [37]. Podocyte detachment leads to podocytopenia which eventually induces glomerulosclerosis. It therefore should be noted that interaction with GBM is the most important and essential role for podocyte survival. Sometimes podocytes encounter mechanical or nonmechanical stress and face disruption of coordinated structure by loss or dysfunction of endogenous components from genetic mutations. No matter what type of stress is given, podocyte foot process effacement can be induced instinctively not to be apart from the GBM and to survive (Figure 2) suggesting that foot process effacement may be the instinct of podocyte to survive.

## 3. Secondary FSGS

Various conditions can cause secondary FSGS (see Table 1). Adaptive response to renal injury leads to renal disease progression in the later stage, diverse drugs and infections can cause glomerular injury and sclerosis directly. In addition, loss or dysfunction of coordinated system to maintain glomerular filtration barrier leads to glomerular sclerosis.

### 3.1. Reduced Renal Mass

Oligomeganephronia, a congenital disease, characterized by larger but fewer glomeruli than normal ones develops FSGS and progresses to chronic renal failure [38]. Vesicoureteral reflux disease is also characterized by reduced renal mass resulting from chronic parenchymal damage and is associated with FSGS [39]. Reduced nephron mass causes glomerular hypertension and hyperfiltration in remaining nephrons. This adaptive mechanism seems to be successful initially but later leads to renal disease progression [40]. As previously discussed, mechanical stretch by glomerular hypertension and hyperfiltration triggers the defense mechanism for podocyte to avoid detachment from GBM and survive. Foot processes of podocyte are effaced to attach the GBM more tightly, and loss of permselectivity of foot process causes proteinuria. However, more important contributing factor to developing glomerular sclerosis is the stimulation of growth which results in endothelial-mesangial hyperplasia and glomerulomegaly [41]. The endothelial-mesangial hyperplasia and glomerulomegaly cause mismatch between tuft growth and podocyte hypertrophy which leads to stretch and attenuation of cell body of podocyte [42]. The loose connection between podocyte and GBM causes podocyte detachment which leads to glomerulosclerosis eventually [15, 43, 44]. Therefore, it should be noted that local soluble factors play an important role in developing FSGS [45]. Several studies demonstrated that mechanical stretch on podocyte increased TGF-*β* and angiotensin II* in vivo* and* in vitro* which promote glomerular hyperfiltration and glomerular growth [15, 44, 46]. Based on these findings, angiotensin blockers are no longer “new”; they are well-proven substances to retard the progression of renal disease [47]. In summary, the significant reduction in number of nephrons such as low birth weight, unilateral renal agenesis, and unilateral nonfunctioning kidney from trauma or vascular insufficiency has a risk for FSGS and progressive renal disease.

### 3.2. Obesity

Obesity-related glomerulopathy (ORG) has generally mild presentations of nephropathy and FSGS is the most common type of ORG. Multiple observations demonstrated the clinical characteristics and outcome of ORG [48]. Obesity-related FSGS has significant amounts of proteinuria but they are less than those of idiopathic FSGS without features of nephrotic syndrome [49]. Most patients with ORG also present with mild and visceral obesity, minor proteinuria, and preserved renal function [50]. The pathologic features of ORG include glomerulomegaly, increased foot process width, decreased podocyte density and number, and global and segmental sclerosis. Particularly, decreased podocyte number is correlated with renal function impairment and also with metabolic disturbances such as glycemia, insulin resistance, and hyperinsulinemia [51]. The hemodynamic and metabolic disturbances are associated with dysregulation of hormones acting on podocytes in ORG [52]. The common features in pathogenesis of ORG include glomerular hyperfiltration, activation of renin-angiotensin-aldosterone system, upregulation of local peptide hormones (angiotensin II and TGF-*β*), insulin resistance and compensatory hyperinsulinemia, and glomerulomegaly [46, 53, 54]. These processes induce oxidative stress, podocyte injury, and apoptosis leading to podocytopenia. Therefore, the drugs blunting those pathways including angiotensin receptor blockers, aldosterone antagonists, and thiazolidinediones may be considered as candidates for the treatment of ORG [49, 53, 55]. A recent study reported a case of obesity-related FSGS, in which 17-year-old girl with obesity-related FSGS unresponsive to medical treatments including angiotensin-converting enzyme inhibitor and steroid and cytotoxic drug showed normalization of proteinuria after bariatric surgery. This observation suggests that body weight reduction is also applicable to improve ORG. Interestingly, normalization of proteinuria was achieved by two weeks after the surgery with 4% reduction of body weight [56].

### 3.3. Drugs

Heroin has been known as a representative drug causing FSGS. However, several studies argued that heroin-induced FSGS was associated with adulterants added to injection and not heroin itself [57]. In epidemiologic study, the incidence of heroin-associated nephropathy was declined as time passed during study periods because purity of heroin was increasing due to reduction of adulterants use [58]. Nevertheless, several studies demonstrated effects of heroin (or morphine) itself on the kidney. Morphine modulates the proliferation of mesangial cell and fibroblasts and expression of slit diaphragm constituting molecules in podocyte [59–61]. In addition, morphine induces oxidative stress in glomerular epithelial cell [62].

### 3.4. Genetic Mutations

Podocyte foot process is maintained by elaborately organized system, which is composed of actin cytoskeleton, synaptopodin, podocalyxin, nephrin, podocin, and so forth. Many genetic mutations cause the dysfunction or loss of foot process components leading to secondary FSGS [19, 63]. An elegant review by D'Agati et al. summarized genetic or familial factors of FSGS [1]. Here, we thus briefly introduce recent progress of gene mutations involving FSGS. Nephrin (*NPHS1*) and podocin (*NPHS2*) are slit diaphragm proteins in podocyte foot process. The patients with genetic mutation of* NPHS1* present with Finnish-type congenital nephrotic syndrome. The mutations of* NPHS1* and* NPHS2* cause nephrotic syndrome resistant to immunotherapy and show less recurrence after renal transplantation [64]. Recently,* APOL1* gene encoding apolipoprotein L1 (ApoL1) issued in the study of an African American has a strong association with FSGS [65]. ApoL1 has the potential to lyse trypanosome causing African trypanosomiasis known as sleeping sickness. The two* APOL1* variants are common in Africa; probably the two gene variants are thought to be evolved to protect Africans against* Trypanosoma brucei*. Recent studies have shown relationships of the two* APOL1* variants with various kidney diseases in Africans or African Americans such as HIV-associated nephropathy (HIV-NP), FSGS, and hypertensive ESRD. Recent studies cumulated the evidences that* APOL1* risk alleles or variants were strongly associated with proteinuric kidney diseases including FSGS [66–68]. However, how the* APOL1* variants act on podocyte to cause FGSG has been ill-defined. Underlying mechanism by which* APOL1* variants regulate podocyte function involving FSGS awaits future investigation.

Additionally, cumulative genetic studies support the fact that genetic mutations play an important role in glomerular diseases including FSGS [69]. The list of genetic mutations causing FSGS probably will continue to grow.

## 4. Conclusion

Secondary FSGS is not a specific disease but a state representing podocyte injury which is mediated by diverse causes including mechanical and/or nonmechanical stresses and genetic mutations. Podocytes interact with GBM and capillary loops tightly, dysfunction of which is an early event leading to FGSG. FSGS seems like a station to stay in just before arriving to destination. Unanswered questions in the pathogenesis of secondary FSGS are still ill-defined. Uncovering the selective targeting to pathogenesis and underlying mechanism of FSGS may provide clues to answer for treatment of the disease in the future.

## Figures and Tables

**Table 1:** Causes of secondary FSGS.

Type	Cause
Adaptive (with reduced renal mass)	Oligomeganephronia, vesicoureteral reflux, low birth weight, unilateral renal agenesis, surgical renal ablation, chronic renal allograft nephropathy

Adaptive (with normal renal mass)	Systemic hypertension, obesity, increased lean body mass, renal vasoocclusive disease, cyanotic congenital heart disease, sickle cell anemia

Drug-induced	Heroin, pamidronate, interferon, lithium, sirolimus

Genetic	*NPHS1, NPHS2, INF2, TRPC6, ACTN4, APOL1*

Virus-associated	HIV-1, parvovirus B19, EBV, CMV
